# Supplementary material for: Activation of TnSmu1, an integrative and conjugative element, by an ImmR-like transcriptional regulator in Streptococcus mutans
Source: Microbiology (Reading). 2022 Oct 6;168(10):001254. doi: 10.1099/mic.0.001254 (PMC10233336; doi:10.1099/mic.0.001254)
Supplement: Supplementary material 1 [file mic-168-1254-s001.pdf]

Supplementary Material for:

Activation of TnSmu1, an integrative and conjugative element, by an ImmR-like transcriptional regulator in *Streptococcus mutans*

Shawn King[1], Allison Quick[1], Kalee King[1], Alejandro R. Walker[2], and Robert C. Shields[1*]
[1] Department of Biological Sciences, Arkansas State University, Jonesboro, Arkansas, USA
[2] Department of Oral Biology, University of Florida, Gainesville, Florida, USA

This PDF file includes:
Figs. S1-S5
Tables S1-S6

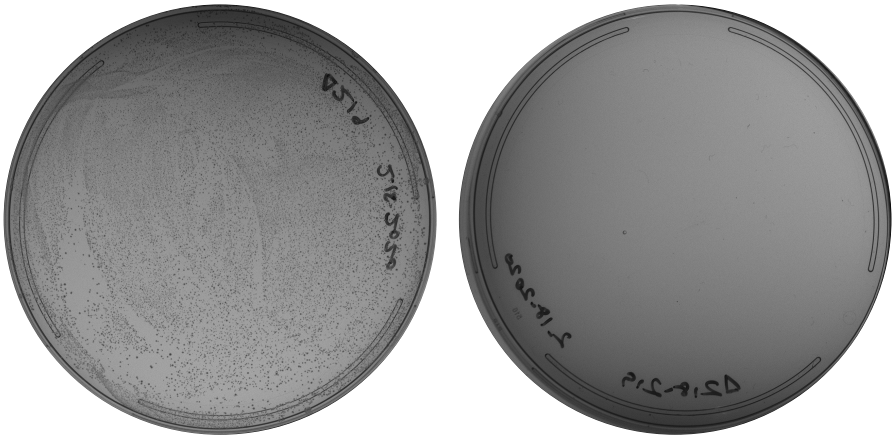

Δ219/*immA*$_{Smu}$ Δ218-219/*immRA*$_{Smu}$

Figure S1. Colony forming units obtained from transformation experiments. For these experiments attempts were made to transform *S. mutans* UA159 with *immA*$_{Smu}$::*aphA-3* or *immRA*$_{Smu}$::*aphA-3*. After transformation, colonies were selected on BHI agar containing kanamycin. Deletion of *immA$_{Smu}$* yielded a significant amount (lawn) of colony forming units. Under the conditions tested, deletion of *immRA*$_{Smu}$ was not permitted by *S. mutans* and zero colonies grew on transformation plates.

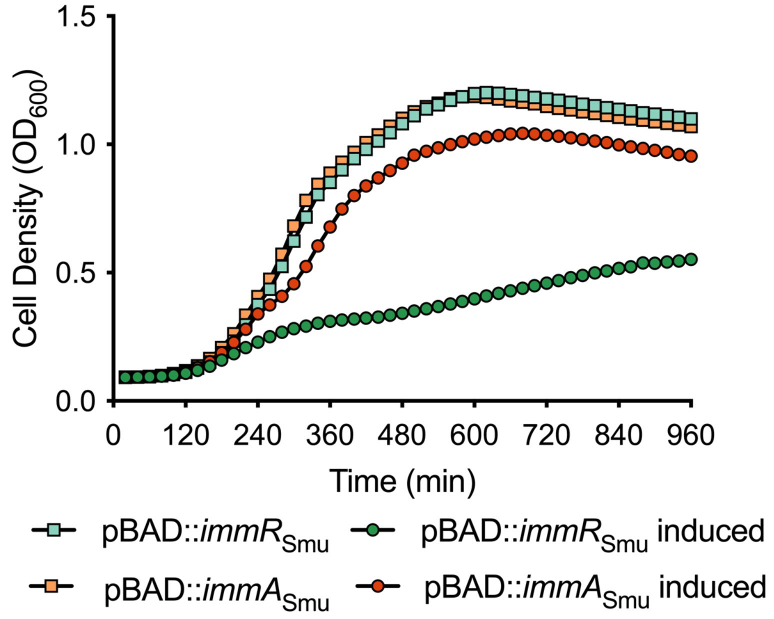

Figure S2. Investigating the impact of *immR*$_{Smu}$ and *immA*$_{Smu}$ expression on *E. coli* growth. Both *immR*$_{Smu}$ and *immA*$_{Smu}$ were cloned into an arabinose inducible protein expression system (pBAD). Induction of ImmA$_{Smu}$ had only a minor impact on *E. coli* growth (red circles). Induction of ImmR$_{Smu}$ (green circles) led to a reduction in the growth rate of *E. coli*, and the final yield after 16 h.

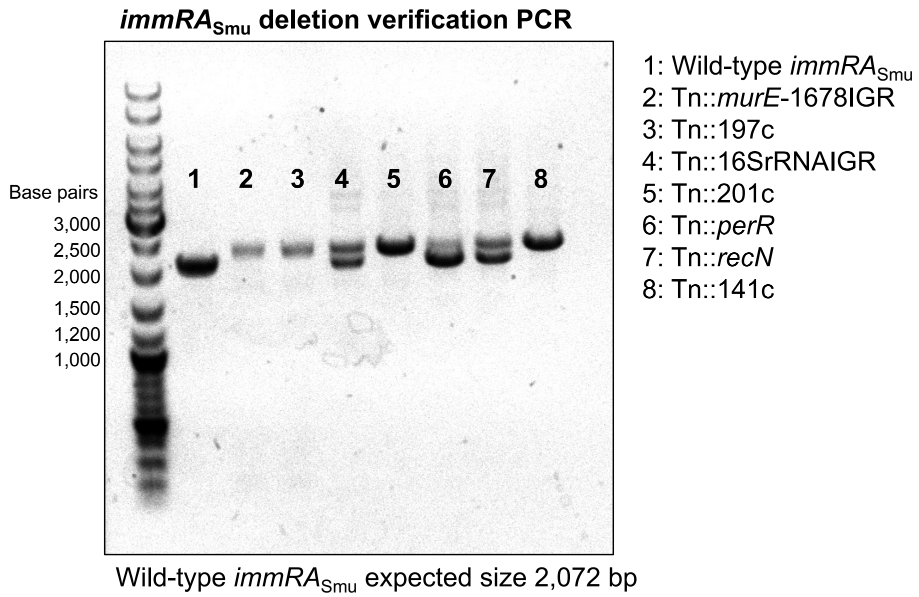

Figure S3. PCR verification of *immRA*$_{Smu}$ mutagenesis. Correct deletion of *immRA*$_{Smu}$ (replacement with the kanamycin resistance gene *aphA3*) was confirmed with PCR. Notably there were mutants with a gene duplication event, as shown by having two PCR products of both a wild-type *immRA*$_{Smu}$ and the expected size for the mutated version.

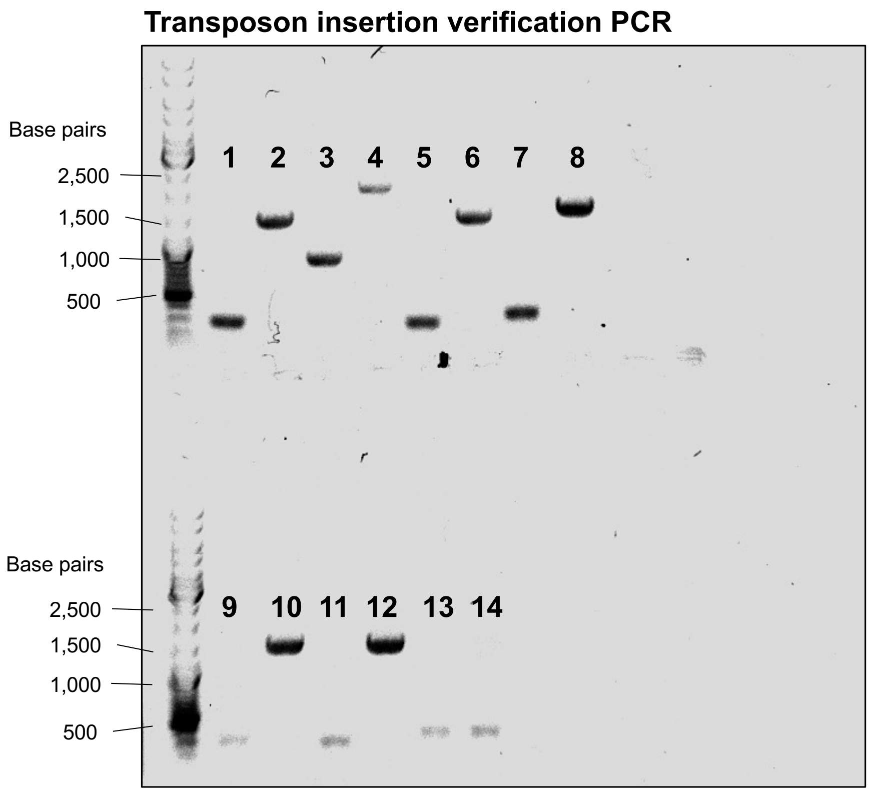

1: Wild-type *murE*-1678IGR amplification
2: Tn::*murE*-1678IGR
3: Wild-type SMU_197c amplification
4: Tn::197c
5: Wild-type 16S rRNA IGR amplification
6: Tn::16SrRNAIGR
7: Wild-type SMU_201c amplification
8: Tn::201c
9: Wild-type perR amplification
10: Tn::*perR*
11: Wild-type recN amplification
12: Tn::*recN*
13: Wild-type SMU_141c amplification
14: Tn::141c

Magellan6 transposon size: 1,127 bp

Figure S4. PCR verification of transposon insertion. Transposon insertions were discovered with genome sequencing and confirmed with PCR. For each expected insertion site, a primer pair was designed to amplify the region. For each mutant strain a wild-type PCR product and a PCR product from the mutant was generated and visualized with gel electrophoresis.

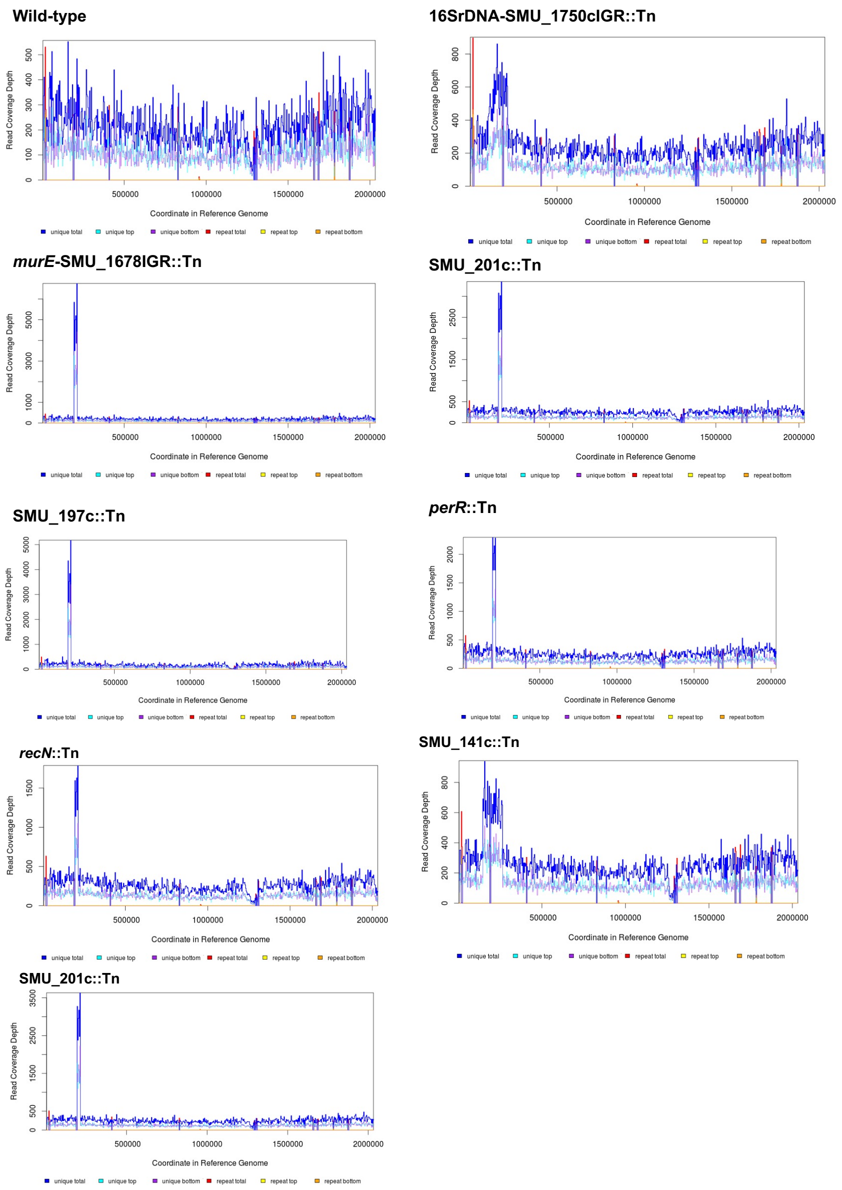

Figure S5. Read coverage depth for sequenced genomes. A notable trend of increased sequencing reads for the TnSmu1 region was observed for most of the double mutant strains (lacking *immRA*$_{Smu}$, or with a gene duplication event).

Table S1. Strains and plasmids used in this study.

Strain	Description	Source
S. mutans strains		
UA159	Wild-type	Burne Lab
Δ*immA*$_{\text{Smu}}$	SMU_219::*aphA3*	This work
Tn-1	IGRmurE_SMU_1678::Tn Δ*immRA*$_{\text{Smu}}$::*aphA3*	This work
Tn-3	SMU_197c::Tn *immRA*$_{\text{Smu}}$::*aphA3*	This work
Tn-7	IGR16SrDNA_SMU_1750c::Tn Δ*immRA*$_{\text{Smu}}$::*aphA3*	This work
Tn-8	SMU_201c::Tn Δ*immRA*$_{\text{Smu}}$::*aphA3*	This work
Tn-9	SMU_593::Tn Δ*immRA*$_{\text{Smu}}$::*aphA3*	This work
Tn-10	SMU_585::Tn Δ*immRA*$_{\text{Smu}}$::*aphA3*	This work
Tn-11	SMU_201c::Tn Δ*immRA*$_{\text{Smu}}$::*aphA3*	This work
Tn-12	SMU_141::Tn Δ*immRA*$_{\text{Smu}}$::*aphA3*	This work
CRISPRi sgRNA-*lacG*	pDL278::P$_{xyl}$-*dcas9* pPM::sgRNA-*lacG*	(1)
CRISPRi sgRNA-*immR*$_{\text{Smu}}$	pDL278::P$_{xyl}$-*dcas9* pPM::sgRNA-*immR*$_{\text{Smu}}$	(1)
E. coli strains		
10-beta	Cloning host, derivative of DH10B	New England Biolabs
BL21(DE3)	Cloning host suitable for protein expression	New England Biolabs
Plasmids		
pBAD/His/A	Protein expression vector with an *araBAD* promoter for tightly regulated expression	Thermo Fisher Scientific

Table S2. Oligonucleotides used in this study.

Name	Oligonucleotide sequence (5'-3')	Description
S. mutans cloning		
219A	TGTTCCCAGAACGCTTAAAA	Deletion of SMU_219
219B	ATGCGGATCCTGATGGTAACTCCAAGTTTTTCTG	Deletion of SMU_219
219C	ATGCGGATCCATGAAAACATTGTTAAAGAAGAAGC	Deletion of SMU_219
219D	TGATGAAGGCAAATTGTGGA	Deletion of SMU_219
219E	TGAGGTTAACTAGATTTTTAACAG	Sequencing deletion of SMU_219
219F	GGAACCTATTATGTTTATAAGTG	Sequencing deletion of SMU_219
218_219A	TCTAGCATGCGCTCATATCCT	Double deletion of SMU_218 and SMU_219
218_219B	ATGCGGATCCTGCTTCTAGGCGCAGAGATT	Double deletion of SMU_218 and SMU_219
218_219E	ACCACTCATCTGCTTAATAA	Sequencing double deletion of SMU_218 and SMU_219
E. coli cloning		
pBAD218Fv2	GATCGGTACCATATGTTCCCAGAACGCTTA	SMU_218 cloning into pBAD/His/A
pBAD218R	GATCGAATTCCGTTTAGTTATTTTTCTGATTTTTGA	SMU_218 cloning into pBAD/His/A
pBAD219Fv2	GATCGGTACCATATGAACTTATCAAAAATTGTTAGAGA	SMU_219 cloning into pBAD/His/A
pBAD219R	GATCGAATTCGTATCTTTTTATTTTGGTTTTGTCA	SMU_219 cloning into pBAD/His/A
pBADseqF	ATGCCATAGCATTTTTATCC	pBAD/His/A sequencing
pBADseqR	GATTTAATCTGTATCAGG	pBAD/His/A sequencing
Transposon verification		
Tn-3_Ins_SeqF	GCGATCACAGAAGCACAAAA	Interaction screen transposon insertion verification
Tn-3_Ins_SeqR	GACAGAGACAAGGCCCAAAA	Interaction screen transposon insertion verification
Tn-1_Ins_SeqF	CGCCAACAGTCGTTAAGGTT	Interaction screen transposon insertion verification
Tn-1_Ins_SeqR	CAGGAGTGTTAGGGATTCCTTG	Interaction screen transposon insertion verification
Tn-7_Ins_SeqF	GACCGAAACTTAGGCTTGGA	Interaction screen transposon insertion verification
Tn-7_Ins_SeqR	TAAAACCCAAGGACGGACTG	Interaction screen transposon insertion verification
Tn-8_Ins_SeqF	AAAGGGACAGGAATCTAGGG	Interaction screen transposon insertion verification
Tn-8_Ins_SeqR	CCTGAAAAAGGAGCCATCTG	Interaction screen transposon insertion verification

Tn-9_Ins_SeqF	TCCTGATTGATGAAGGCTTTG	Interaction screen transposon insertion verification
Tn-9_Ins_SeqR	TGACTCACCTCCTATTTCCCATA	Interaction screen transposon insertion verification
Tn-10_Ins_SeqF	CGGTAAATGACCTCGCTTTT	Interaction screen transposon insertion verification
Tn-10_Ins_SeqR	TGAGACGAGACAGCTCTCCA	Interaction screen transposon insertion verification
Tn-12_Ins_SeqF	GGCTGCTCAGACAGGAAATC	Interaction screen transposon insertion verification
Tn-12_Ins_SeqR	CTCACACCAAGCAATGATGG	Interaction screen transposon insertion verification
TnSmu1 qPCR		
CircTnSmu1.1F	AAATTTTCTCCCAAAAATTATCAAA	qPCR for circular TnSmu1
CircTnSmu1.1R	AAAGAGTTTAAAGAGGTTGAACAAA	qPCR for circular TnSmu1
ExcisTnSmu1F	CAATTCCTACTGCCCGTGTT	qPCR for TnSmu1 excision
ExcisTnSmu1R	GATATTTGGGCGGTTGCTAA	qPCR for TnSmu1 excision
TnSmu1sloRF	TTTATCGCAAGCATCGTCTG	qPCR for chromosomal gene
TnSmu1sloRR	GGCTGTCCATGTTGAGGAAT	qPCR for chromosomal gene

Table S3. Transcriptomic changes measured by RNA-seq when *immR*$_{Smu}$ is repressed by CRISPRi

Gene ID	Gene name	Annotation	K number	COG	Log2 fold change	FDR
SMU_204c	NA	hypothetical protein	no	-	10.03	3.28E-06
SMU_211c	NA	hypothetical protein	no	-	8.92	8.74E-06
SMU_201c	NA	conserved hypothetical protein	no	-	8.78	5.50E-06
SMU_209c	NA	hypothetical protein	no	-	8.76	3.78E-06
SMU_202c	NA	conserved hypothetical protein/Streptococcus-specific protein	no	-	8.33	6.02E-06
SMU_197c	NA	hypothetical protein	no	-	8.05	1.10E-08
SMU_206c	NA	hypothetical protein	no	-	7.97	3.41E-05
SMU_199c	NA	hypothetical protein	no	-	7.76	8.99E-06
SMU_213c	NA	hypothetical protein	no	-	7.60	1.69E-05
SMU_210c	NA	hypothetical protein	no	-	7.51	1.74E-06
SMU_208c	NA	conserved hypothetical protein, FtsK/SpoIIIE family	no	-	7.51	1.10E-08
SMU_198c	tpn	conjugative transposon protein	no	-	7.26	9.96E-08
SMU_200c	NA	hypothetical protein	no	-	7.26	8.87E-06
SMU_205c	NA	conserved hypothetical protein	no	-	7.06	1.77E-05
SMU_196c	NA	immunogenic secreted protein (transfer protein)	no	-	6.70	1.16E-08
SMU_207c	NA	transcriptional regulator	no	-	6.64	2.85E-07
SMU_216c	NA	hypothetical protein	no	-	5.66	2.00E-04
SMU_214c	NA	hypothetical protein	no	-	5.61	2.00E-04
SMU_212c	NA	hypothetical protein	no	-	5.45	5.04E-04
SMU_221c	NA	phage-related integrase, truncated	no	-	5.24	1.08E-06
SMU_195c	NA	hypothetical protein	no	-	4.83	4.95E-05
SMU_217c	NA	conserved hypothetical protein; Streptococcus-specific protein	no	-	4.75	4.37E-05
SMU_215c	NA	hypothetical protein	no	-	4.53	5.04E-04
SMU_934	NA	amino acid ABC transporter, permease protein	K16958	E	4.31	1.14E-07
SMU_194c	NA	conserved hypothetical protein, phage-related	no	-	4.24	2.00E-04
SMU_962	mmgC	acyl-CoA dehydrogenase	no	I	4.01	1.24E-07
SMU_219	NA	hypothetical protein	no	-	3.99	1.54E-06
SMU_1753c	cas2	CRISPR-associated endoribonuclease Cas2	K09951	L	3.95	3.05E-07

SMU_936	NA	amino acid ABC transporter, ATP-binding protein	K16960	E	3.76	1.49E-06
SMU_220c	NA	hypothetical protein	no	-	3.67	3.78E-06
SMU_1155	NA	hypothetical protein	no	-	3.67	1.54E-02
SMU_1757c	cas1	CRISPR-associated protein Cas1	no	-	3.61	6.02E-06
SMU_1036	NA	conserved hypothetical protein	K01992	V;R;C;P;G;M	3.56	4.16E-05
SMU_1755c	cas1	CRISPR-associated protein Cas1	no	-	3.53	4.76E-06
SMU_1889c	NA	hypothetical protein (possible relation to bacteriocin BlpU)	no	-	3.51	1.67E-05
SMU_193c	NA	conserved hypothetical protein	no	-	3.49	5.97E-04
SMU_1900	comB	ABC transporter	K20345	-	3.48	2.99E-05
SMU_961	NA	macrophage infectivity potentiator-related protein	no	S	3.46	1.18E-06
SMU_218	NA	transcriptional regulator	no	-	3.45	4.43E-05
SMU_653c	tauC	ABC transporter, permease protein (possible taurine transport system permease)	K02050	P	3.43	7.80E-06
SMU_574c	lrg	effector of murein hydrolase	K05339	M	3.29	5.39E-03
SMU_1763c	cas5	CRISPR-associated protein, Cas5d-type	K19119	-	3.24	1.69E-05
SMU_932	NA	conserved hypothetical protein	no	T	3.22	2.95E-05
SMU_935	NA	amino acid ABC transporter, permease protein	K16959	E	3.19	4.47E-06
SMU_1154c	NA	conserved hypothetical protein	no	-	3.15	3.15E-05
SMU_1758c	cas4	CRISPR-associated protein Cas4	K07464	L	3.04	1.74E-06
SMU_656	mutE2	ABC transporter, permease, possibly bacteriocin associated	K20491	-	2.99	8.42E-03
SMU_933	atmA	amino acid ABC transporter, amino acid substrate-binding protein	K16957	E;T	2.96	1.69E-05
SMU_1029	NA	hypothetical - transposon	no	-	2.89	2.02E-03
SMU_1156c	NA	hypothetical protein	no	-	2.88	1.06E-02

SMU_652c	msmK	ABC transporter, ATP-binding protein (possible nitrate transport system)	K15555	P	2.84	1.14E-04
SMU_1754c	cas1	CRISPR-associated protein Cas1	no	-	2.83	4.62E-06
SMU_59	asl purB	adenylosuccinate lyase	K01756	F	2.79	1.34E-03
SMU_1762c	csd1	CRISPR-associated protein, Csd1-type	no	-	2.77	1.22E-02
SMU_1410	frdC	fumarate reductase	K00244	R	2.77	1.31E-04
SMU_1598	celC licA	PTS system IIA component, required for cellobiose uptake and metabolism	K02759	G	2.74	2.15E-02
SMU_50	purE	phosphoribosylaminoimidazole carboxylase catalytic subunit	K01588	F	2.66	2.28E-04
SMU_350	NA	hypothetical protein	no	-	2.61	8.10E-04
SMU_51	purK	phosphoribosylaminoimidazole carboxylase, ATPase subunit	K01589	F	2.51	8.52E-04
SMU_1760c	csd2	CRISPR-associated protein Csd2	K19118	-	2.50	2.15E-05
SMU_1322	butA	acetoin reductase	K03366	I;Q;R	2.45	1.14E-04
SMU_132	hipO	amino acid amidohydrolase (hippurate amidohydrolase)	no	R	2.41	1.74E-05
SMU_441	NA	transcriptional regulator	no	K	2.38	2.00E-04
SMU_113	pfk	fructose-1-phosphate kinase	K00882	G	2.38	3.34E-02
SMU_442	NA	conserved hypothetical protein	no	-	2.36	3.87E-04
SMU_48	purD	phosphoribosylamine-glycine ligase	K01945	F	2.34	9.19E-04
SMU_1981c	comG comGF	competence protein G	K02248	U	2.34	4.77E-02
SMU_145	NA	major facilitator superfamily transporter, efflux protein	no	P	2.32	6.40E-04
SMU_1218	gatA	Gln-dependent amidotransferase, subunit A	K01426	J	2.29	3.35E-05
SMU_666	argD	N-acetylornithine aminotransferase	K00821	E	2.28	2.28E-04
SMU_1938c	atmE	amino acid ABC transport permease	K02072	P	2.27	5.87E-05

SMU_662	NA	conserved hypothetical protein (possible membrane protein)	K07052	-	2.24	3.46E-05
SMU_94c	tpn	transposase fragment	no	-	2.23	5.38E-03
SMU_1970c	pheT syfB	phenylalanyl-tRNA synthetase beta subunit	K06878	R	2.21	1.94E-03
SMU_1761c	csd1	CRISPR-associated protein, Csd1-type	K19117	L	2.17	3.37E-04
SMU_1319c	NA	conserved hypothetical protein	no	S	2.16	5.97E-04
SMU_1578	birA	biotin--[acetyl-CoA-carboxylase] ligase	K03524	H	2.14	8.91E-04
SMU_1486c	NA	histidinol phosphatase	K04486	E;R	2.13	1.48E-04
SMU_1356c	tpn	transposase	no	-	2.12	3.16E-03
SMU_658	NA	conserved hypothetical protein	no	-	2.12	5.75E-04
SMU_1207	fic mobC	cell filamentation / mobilization protein	K04095	D	2.12	3.21E-05
SMU_2119	opcD opuCD	ABC transport betaine/carnitine/choline permease	K05846	E	2.11	8.45E-04
SMU_911c	NA	hypothetical protein	no	-	2.10	2.06E-04
SMU_1405c	NA	conserved hypothetical protein	K09952	S	2.10	5.85E-05
SMU_1764c	cas3	CRISPR-associated helicase	K07012	-	2.09	9.82E-05
SMU_92c			no	-	2.09	2.26E-02
SMU_1770	syv valS	valyl-tRNA synthetase	K01873	J	2.08	1.57E-03
SMU_1095	atmE pstA pstC	proline/glycine betaine ABC permease and solute binding protein	K05845	M	2.08	3.40E-04
SMU_496	cysK	cysteine synthetase A	K01738	E	2.07	1.28E-04
SMU_349	ksgA	dimethyladenosine transferase	K02528	J	2.07	5.97E-04
SMU_1658	nrgA	ammonium transporter, NrgA protein	K03320	P	2.07	4.98E-04
SMU_1897	NA	ABC transporter, ATP-binding protein; similar to BlpA	no	-	2.06	8.09E-04
SMU_1548c	hk11 yvfT	sensor histidine kinase	K07778	T	2.03	2.01E-04
SMU_1592	pepQ	proline dipeptidase	K01271	E	2.03	1.61E-04
SMU_657	mutG	ABC transporter, permease, possibly bacteriocin associated	K20492	-	2.03	1.54E-02

SMU_872	fruA fxpC	fructose-specific PTS system enzyme IIBC component	K02768	G	2.02	1.95E-03
SMU_2038	pttB treB	phosphotransferase system, trehalose-specific IIBC component (EIIBC-tre)	K02817	G	2.02	4.95E-05
SMU_1216c	NA	ABC transporter, amino acid permease	K10009	E	2.00	9.46E-04
SMU_1203	bcat ilvE	branched-chain amino acid aminotransferase	K00826	E;H	2.00	5.97E-04
SMU_1257c	NA	conserved hypothetical protein	no	S	2.00	2.47E-04
SMU_1343c	pksC pksL	polyketide synthase	no	-	-2.05	8.48E-03
SMU_2019	rl29 rpmC	50s ribosomal protein L29	K02904	J	-2.05	5.99E-04
SMU_1059	satC	acid tolerance protein	no	-	-2.09	4.01E-03
SMU_748	NA	hypothetical protein	no	-	-2.12	2.54E-02
SMU_2018	rpsQ rs17	30S ribosomal protein S17	K02961	J	-2.13	4.95E-05
SMU_420	NA	ribosomal protein L7A family	no	J	-2.13	5.39E-03
SMU_1344c	fabD	malonyl CoA-acyl carrier protein transacylase	no	I	-2.14	1.59E-02
SMU_1061	ylxM	DNA-binding protein	K09787	S	-2.17	7.57E-03
SMU_2012	rpsH rs8	30S ribosomal protein S8	K02994	J	-2.25	2.04E-04
SMU_451	NA	hypothetical protein	no	-	-2.29	1.46E-02
SMU_758c	NA	conserved hypothetical protein	no	-	-2.32	3.07E-04
SMU_547	NA	conserved hypothetical protein	no	-	-2.32	2.47E-04
SMU_276c	NA	hypothetical protein	no	-	-2.34	3.82E-02
SMU_27	ACP acpP	acyl carrier protein	K02078	I;Q	-2.34	1.63E-03
SMU_866	NA	conserved hypothetical protein	K06960	R	-2.35	4.74E-05
SMU_1340	bacA	bacitracin synthetase 1/ tyrocidin synthetase III	no	Q	-2.36	3.37E-04
SMU_941c	NA	conserved hypothetical protein	no	-	-2.39	1.05E-04
SMU_541	NA	conserved hypothetical protein	no	S	-2.39	1.82E-02
SMU_29	purC	phosphoribosylaminoimidazole-succinocarboxamide synthase	K01923	F	-2.47	3.24E-03
SMU_1643c	NA	conserved hypothetical protein	no	-	-2.47	2.19E-02

SMU_1610	rpmG	50S ribosomal protein L33	K02913	J	-2.48	3.06E-02
SMU_1127	rpsT	30S ribosomal protein S20	K02968	J	-2.50	5.97E-04
SMU_18	NA	hypothetical protein	no	-	-2.51	3.41E-02
SMU_768c	NA	conserved hypothetical protein	no	-	-2.52	2.25E-02
SMU_1339	bacC	bacitracin synthetase; surfactin synthetase	no	Q	-2.53	4.33E-04
SMU_1671c	NA	conserved hypothetical protein	no	S	-2.54	4.52E-03
SMU_1902c	NA	hypothetical protein	no	-	-2.56	7.13E-03
SMU_628	holA	DNA polymerase III, delta subunit	K02340	L	-2.65	1.63E-03
SMU_1345c	ituA mycA	peptide synthetase similar to mycA	no	I;Q	-2.68	9.16E-04
SMU_1507c	NA	hypothetical protein	no	-	-2.72	3.31E-02
SMU_1907	NA	hypothetical protein	no	-	-2.77	3.22E-02
SMU_637c	NA	hypothetical protein	no	-	-2.78	4.65E-04
SMU_722	NA	hypothetical protein	no	-	-3.01	1.15E-02
SMU_545	NA	hypothetical protein	no	-	-3.77	2.73E-02

Table S4. Location and analysis of *immR*$_{Smu}$ transposon double mutant strains.

Name	Insertion Site	Genomic Observations	Δ*erfRP**	Tn Insertion
**Tn-1**	IGR between *murE* and SMU_1678	New junction SMU_191c and SMU_220c	Yes	Yes
**Tn-3**	SMU_197c	New junction SMU_191c and SMU_220c	Yes	Yes
**Tn-7**	IGR between 16S rDNA and SMU_1750c	n/a	GD	Yes
**Tn-8**	SMU_201c	New junction SMU_191c and SMU_220c	Yes	Yes
**Tn-9**	*furR* (SMU_593)	New junction SMU_191c and SMU_220c	GD	Yes
**Tn-10**	*recN* (SMU_585)	New junction SMU_191c and SMU_220c	GD	Yes
**Tn-11**	SMU_201c	New junction SMU_191c and SMU_220c	Yes	Yes
**Tn-12**	SMU_141	New junction *mleS*-*sgaT* / T268T SMu.351 GTPase	Yes	Inconclusive

*GD, gene duplication event

Table S5. RNA-seq analysis of SMU_197c::Tn Δ*immR*$_{Smu}$ compared with *S. mutans* UA159

Gene ID	Gene Name	Annotation	Log_2 fold change	FDR
SMU_205c		hypothetical protein	8.64	0
SMU_198c		putative conjugative transposon protein	8.60	0
SMU_211c		hypothetical protein	8.30	0
SMU_215c		hypothetical protein	8.21	0
SMU_206c		hypothetical protein	8.19	0
SMU_213c		hypothetical protein	8.15	0
SMU_199c		hypothetical protein	8.11	6.665e-321
SMU_204c		hypothetical protein	8.08	0
SMU_212c		hypothetical protein	8.06	0
SMU_217c		hypothetical protein	8.03	0
SMU_216c		hypothetical protein	8.03	2.79E-288
SMU_210c		hypothetical protein	8.00	0
SMU_207c		putative transposon protein	7.95	0
SMU_209c		hypothetical protein	7.95	0
SMU_214c		hypothetical protein	7.95	3.91E-192
SMU_208c		putative transposon protein; possible DNA segregation ATPase	7.84	0
SMU_200c		hypothetical protein	7.59	7.37E-306
SMU_202c		hypothetical protein	7.38	7.53816e-319
SMU_201c		putative transposon protein	6.86	0
SMU_197c		hypothetical protein	6.80	0
SMU_1754c	*cas1*	CRISPR-associated protein Cas1	4.21	6.49E-178
SMU_1753c	*cas2*	CRISPR-associated endoribonuclease Cas2	4.14	5.74E-296
SMU_1760c	*csd2*	CRISPR-associated protein Csd2	4.07	4.56E-236
SMU_1761c	*csd1*	CRISPR-associated protein, Csd1-type	4.06	1.52E-305
SMU_1758c	*cas4*	CRISPR-associated protein Cas4	4.04	5.92E-201
SMU_1762c	*csd1*	CRISPR-associated protein, Csd1-type	4.02	1.43E-206
SMU_1755c	*cas1*	CRISPR-associated protein Cas1	3.98	4.82E-163
SMU_1763c	*cas5*	CRISPR-associated protein, Cas5d-type	3.96	3.80E-304
SMU_1757c	*cas1*	CRISPR-associated protein Cas1	3.93	9.15E-173
SMU_196c		putative transfer protein	3.89	1.41E-185

SMU_1764c	*cas3*	CRISPR-associated helicase	3.88	5.92E-293
SMU_194c		conserved hypothetical protein; Bacteriophage P2 associated	3.62	1.07E-107
SMU_191c		putative integrase	3.62	1.31E-151
SMU_1752c		hypothetical protein	3.61	3.70E-197
SMU_195c		hypothetical protein	3.61	5.13E-151
SMU_1750c		hypothetical protein	3.56	8.01E-124
SMU_193c		conserved hypothetical protein	3.44	1.10E-79
SMU_220c		hypothetical protein	3.35	4.83E-113
SMU_1898		putative ABC transporter, ATP-binding and permease protein	2.90	1.01E-95
SMU_1899		putative ABC transporter, ATP-binding and permease protein (fragment)	2.85	3.91E-29
SMU_40		conserved hypothetical protein	2.69	4.37E-12
SMU_41		hypothetical protein	2.52	1.36E-09
SMU_1000		hypothetical protein	2.51	3.67E-22
SMU_1900	*comB*	conserved hypothetical protein	2.46	9.23E-49
SMU_1597c		conserved hypothetical protein	2.18	2.71E-09
SMU_113		putative fructose-1-phosphate kinase	2.10	1.73E-20
SMU_1598	*celC*	putative PTS system, cellobiose-specific IIA component	2.06	6.61247E-06
SMU_941c		conserved hypothetical protein	-2.07	1.14E-68
SMU_924	*tpx*	thiol peroxidase	-2.29	1.07E-32
SMU_140	*gshR*	putative glutathione reductase	-3.40	4.31E-32
SMU_139	*oxdC*	conserved hypothetical protein	-3.46	8.84E-32
SMU_141		conserved hypothetical protein	-3.46	6.11E-32
SMU_137	*mleS*	malolactic enzyme	-3.68	4.13E-31
SMU_138	*mleP*	putative malate permease	-4.11	1.34E-32

Table S6. RNA-seq analysis of SMU_201c::Tn Δ*immR*$_{Smu}$ compared with *S. mutans* UA159

Gene ID	Gene Name	Annotation	Log_2 fold change	FDR
SMU_205c		hypothetical protein	9.92	0
SMU_204c		hypothetical protein	9.55	0
SMU_202c		hypothetical protein	8.48	0
SMU_212c		hypothetical protein	8.38	0
SMU_215c		hypothetical protein	8.36	0
SMU_213c		hypothetical protein	8.27	0
SMU_211c		hypothetical protein	8.26	0
SMU_206c		hypothetical protein	8.17	0
SMU_216c		hypothetical protein	8.01	6.43E-287
SMU_214c		hypothetical protein	8.00	6.13E-194
SMU_217c		hypothetical protein	7.97	0
SMU_209c		hypothetical protein	7.95	0
SMU_210c		hypothetical protein	7.85	0
SMU_208c		putative transposon protein; possible DNA segregation ATPase	7.64	0
SMU_201c		putative transposon protein	7.60	0
SMU_207c		putative transposon protein	7.45	0
SMU_197c		hypothetical protein	4.80	6.03E-253
SMU_198c		putative conjugative transposon protein	4.75	0
SMU_195c		hypothetical protein	4.70	3.51E-242
SMU_200c		hypothetical protein	4.70	7.29E-143
SMU_196c		putative transfer protein	4.69	9.50E-256
SMU_191c		putative integrase	4.65	1.54E-232
SMU_199c		hypothetical protein	4.50	2.24E-129
SMU_194c		conserved hypothetical protein; Bacteriophage P2 associated	4.49	2.77E-158
SMU_193c		conserved hypothetical protein	4.17	3.00E-113
SMU_1753c	*cas2*	CRISPR-associated endoribonuclease Cas2	3.91	8.56E-269
SMU_1754c	*cas1*	CRISPR-associated protein Cas1	3.88	6.10E-155
SMU_1757c	*cas1*	CRISPR-associated protein Cas1	3.79	1.61E-162

SMU_1755c	*cas1*	CRISPR-associated protein Cas1	3.76	1.96E-147
SMU_1762c	*csd1*	CRISPR-associated protein, Csd1-type	3.75	1.57E-183
SMU_1761c	*csd1*	CRISPR-associated protein, Csd1-type	3.75	3.91E-267
SMU_1763c	*cas5*	CRISPR-associated protein, Cas5d-type	3.72	7.08E-273
SMU_1758c	*cas4*	CRISPR-associated protein Cas4	3.71	1.04E-173
SMU_1760c	*csd2*	CRISPR-associated protein Csd2	3.70	2.56E-200
SMU_1764c	*cas3*	CRISPR-associated helicase	3.69	1.54E-267
SMU_1752c		hypothetical protein	3.56	3.43E-192
SMU_1750c		hypothetical protein	3.40	3.66E-114
SMU_220c		hypothetical protein	3.15	3.13E-101
SMU_40		conserved hypothetical protein	2.65	1.25E-11
SMU_1029		conserved hypothetical protein	2.55	5.63E-17
SMU_41		hypothetical protein	2.41	1.13E-08
SMU_1899		putative ABC transporter, ATP-binding and permease protein (fragment)	2.29	9.73E-19
SMU_1898		putative ABC transporter, ATP-binding and permease protein	2.18	1.37E-55
SMU_1539		putative 1,4-alpha-glucan branching enzyme	-2.19	4.59E-58
SMU_1538		putative glucose-1-phosphate adenylyltransferase; ADP-glucose pyrophosphorylase	-2.26	1.36E-63
SMU_141		conserved hypothetical protein	-3.55	3.30E-33
SMU_140	*gshR*	putative glutathione reductase	-3.79	4.47E-38
SMU_139	*oxdC*	conserved hypothetical protein	-3.82	3.70E-37
SMU_137	*mleS*	malolactic enzyme	-4.18	8.59E-38
SMU_138	*mleP*	putative malate permease	-4.44	1.24E-36

References

1. Shields RC, Walker AR, Maricic N, Chakraborty B, Underhill SAM, Burne RA. 2020. Repurposing the *Streptococcus mutans* CRISPR-Cas9 System to Understand Essential Gene Function. PLoS Pathog 16:e1008344.
